# Air Pollution and Atopic Dermatitis (AD): The Impact of Particulate Matter (PM_10_) on an AD Mouse-Model

**DOI:** 10.3390/ijms21176079

**Published:** 2020-08-24

**Authors:** Yu Ri Woo, Seo-Yeon Park, Keonwoo Choi, Eun Sun Hong, Sungjoo Kim, Hei Sung Kim

**Affiliations:** 1 Department of Dermatology, Incheon St. Mary’s Hospital, The Catholic University of Korea, Seoul 06591, Korea; w1206@naver.com (Y.R.W.); dmsun@naver.com (E.S.H.); 2 Department of Biomedicine & Health Sciences, The Catholic University of Korea, Seoul 06591, Korea; gkrud777@gmail.com (S.-Y.P.); icsoo123@naver.com (K.C.); sjkyoon@catholic.ac.kr (S.K.)

**Keywords:** air pollution, atopic dermatitis, particulate matter, AD mouse model, genetic analysis

## Abstract

Air pollution reportedly contributes to the development and exacerbation of atopic dermatitis (AD). However, the exact mechanism underlying this remains unclear. To examine the relationship between air pollution and AD, a clinical, histological, and genetic analysis was performed on particulate matter (PM)-exposed mice. Five-week-old BALB/c mice were randomly divided into four groups (control group, ovalbumin (OVA) group, PM group, OVA + PM group; *n* = 6) and treated with OVA or PM_10,_ alone or together. Cutaneous exposure to OVA and PM_10_ alone resulted in a significant increase in skin severity scores, trans-epidermal water loss (TEWL) and epidermal thickness compared to the control group at Week 6. The findings were further accentuated in the OVA + PM group showing statistical significance over the OVA group. A total of 635, 501, and 2149 genes were found to be differentially expressed following OVA, PM_10_, and OVA + PM_10_ exposure, respectively. Strongly upregulated genes included *RNASE2A*, *S100A9*, *SPRR2D*, *THRSP*, *SPRR2A1* (OVA vs. control), *SPRR2D*, *S100A9*, *STFA3*, *CHIL1*, *DBP*, *IL1B* (PM vs. control) and *S100A9*, *SPRR2D*, *SPRR2B*, *S100A8*, *SPRR2A3* (OVA + PM vs. control). In comparing the groups OVA + PM with OVA, 818 genes were differentially expressed with *S100A9*, *SPRR2B*, *SAA3*, *S100A8*, *SPRR2D* being the most highly upregulated in the OVA + PM group. Taken together, our study demonstrates that PM_10_ exposure induces/aggravates skin inflammation via the differential expression of genes controlling skin barrier integrity and immune response. We provide evidence on the importance of public awareness in PM-associated skin inflammation. Vigilant attention should be paid to all individuals, especially to those with AD.

## 1. Introduction

Air pollution is an important environmental issue and a major threat to global health [1]. Particulate matter (PM), a key component of air pollution, is a designated carcinogen [2], and is well known to increase the risk of cardiovascular and respiratory diseases [3,4]. In recent years, the damaging effect of PM on the skin has raised great interest [5,6,7].

Atopic dermatitis (AD) is an inflammatory, chronically relapsing, and intensely pruritic skin condition. With a prevalence of 2 to 5% (approximately 15% in children and young adults), it is one of the most common skin diseases in industrialized countries. AD has a strong genetic predisposition, but its recent surge in incidence also stresses the role of the environment in the pathogenesis of AD. According to epidemiologic studies, air pollution/PM significantly influences the symptoms of AD [1,8,9,10,11,12,13,14]. However, there is little definitive mechanistic evidence supporting this [15].

PM is a heterogenous mixture of solid and liquid particles suspended in air. Based on particle size, PM is categorized into PM_0.1_ (ultrafine particles, ≤0.1 μm), PM_2.5_ (fine particles, ≤2.5 μm), and PM_10_ (inhalable particles, ≤10 μm). PM_10_ encompasses PM_2.5_ and varies in composition depending on the source [16]. For this study, we employed a standard reference material^®^ 2787 (SRM 2787), issued by the National Institutes of Standards and Technology (NIST). The SRM 2787 is “natural” (field collected) PM composed of polycyclic aromatic hydrocarbons (PAHs), nitro-PAHs, polybrominated diphenyl ethers (PBDEs), dioxins, sugars, and trace elements (i.e., Hg, Al, Ca, Cu, Fe, Pb, Mn, Mg, Ni), with a mean particle diameter of ≤10 μm [17].

As an important interface with the outside environment, the skin, along with the oral and respiratory routes, is a common pathway, through which ambient pollutants enter the body [18,19]. With that said, the potential mechanisms by which PM_10_ exerts cutaneous detrimental effects include direct insult by localization (adherence or penetration of PM to the skin) and indirect injury by systemic inflammation and oxidative stress (i.e., systemic increase of reactive oxygen species (ROS) through the respiratory system) [7].

Taking into account that epithelial barrier dysfunction and cutaneous inflammation are crucial in the pathogenesis of AD [20], the aim of the present work was to evaluate the ability of topically delivered PM to clinically promote AD, and to assess the mechanisms involved in this process by gene analysis (i.e., focusing on genes associated with skin barrier function and the inflammatory pathway). To approximate the condition of AD, we used ovalbumin (OVA)-challenged mice as the animal model.

## 2. Results

### 2.1. Gross Observation and Physiologic Parameters

**Figure 1:**
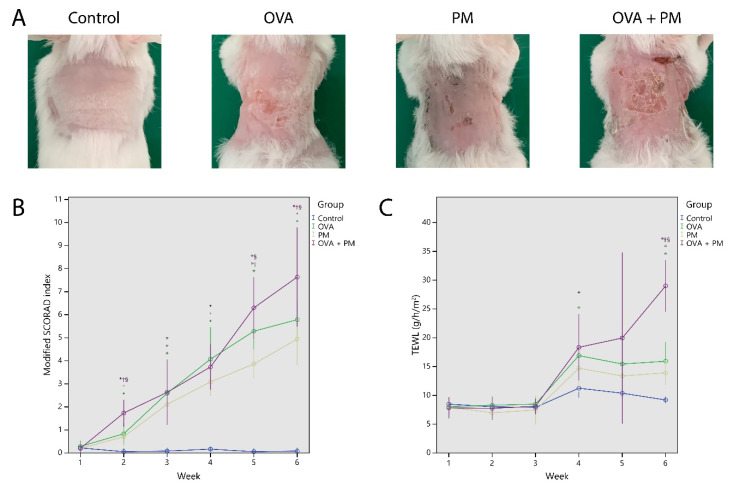
(**A**) Macroscopic findings of the dorsal skin of mice. (**B**) The modified scoring atopic dermatitis (SCORAD) index. (**C**) The trans-epidermal water loss (TEWL). * *p* < 0.05 compared to control, † *p* < 0.05 compared to OVA, § *p* < 0.05 compared to PM_10_.

Repeated topical application of OVA to the dorsal skin (2 × 2 cm) of BALB/c mice induced AD-like skin lesions with erythema, edema, excoriation, and scaling (Figure 1A). The skin severity score at Week 6 was higher in the OVA group (5.79 ± 0.91) and the PM group (4.94 ± 1.08) compared to control (0.08 ± 0.14) (*p* < 0.01). The skin severity score of the OVA + PM group (7.63 ± 1.73) was significantly higher than that of the OVA group and the PM group (*p* < 0.05). The skin severity score was similar between the OVA group and the PM group (*p* > 0.05) (Figure 1B).

OVA (OVA group) and PM_10_ (PM group) application caused an increase in trans-epidermal water loss (TEWL). TEWL at Week 6 was significantly higher in the OVA group (15.9 ± 3.66) and the PM group (13.9 ± 1.99), compared to control (9.20 ± 0.56) (*p* < 0.01). TEWL of the OVA + PM group (29.0 ± 3.61) was significantly higher than that of the OVA group and the PM group at Week 6 (*p* < 0.01). The TEWL was similar between the OVA and the PM group (*p* > 0.05) (Figure 1C).

### 2.2. Hisopathologic Findings

**Figure 2:**
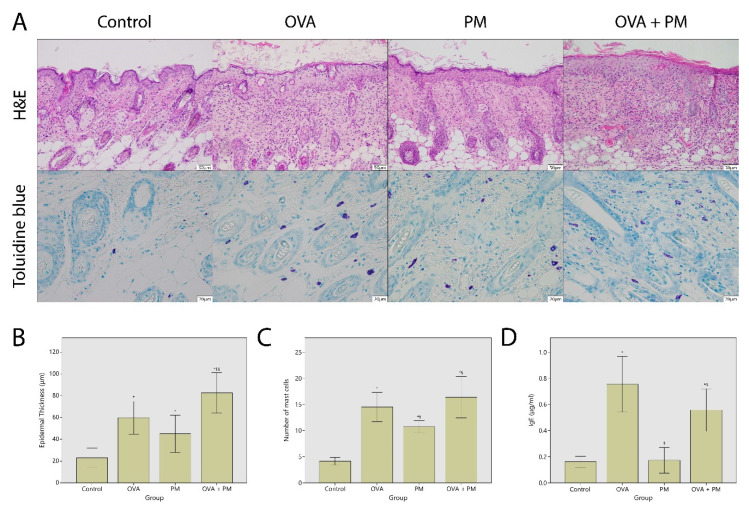
(**A**) (upper) Histologic effects of ovalbumin (OVA) and particulate matter (PM)_10_ on the back skin of mice (hematoxylin and eosin (H&E); ×100), (lower) mast cell infiltration following OVA and PM_10_ exposure (toluidine blue; ×400). (**B**) Epidermal thickness. (**C**) The number of mast cells in five randomly chosen high power fields at ×400 magnification. (**D**) Serum IgE. * *p* < 0.05 compared to control, † *p* < 0.05 compared to OVA, § *p* < 0.05 compared to PM_10_.

Figure 2A demonstrates the hematoxylin and eosin (H&E) and toluidine blue staining of the dorsal skin. There was marked epidermal thickening after OVA (OVA group) and PM_10_ (PM group) application at Week 6 compared to control (59.8 ± 16.3 and 45.1 ± 16.3 μm vs. 23.2 ± 8.42 μm) (*p* < 0.05). The epidermis of the OVA + PM group (82.6 ± 15.0) was significantly thicker than that of the OVA group and the PM group at Week 6 (*p* < 0.05). The epidermal thickness was similar between the OVA and the PM group (*p* > 0.05) (Figure 2B).

As shown in Figure 2A, OVA and PM_10_ increased mast cell infiltration in the dermis. The mast cell number was significantly higher in the OVA group (14.5 ± 3.04/5 high power fields) and the PM group (10.7 ± 1.10), compared to control (4.17 ± 0.72) (*p* < 0.01). The number of mast cells in OVA treated groups (the OVA group, the OVA + PM group: 16.4 ± 3.19/5 high power fields) were significantly higher than that of the PM treated group (*p* < 0.05). The mast cell number was similar between the OVA + PM and the OVA group (*p* > 0.05) (Figure 2C).

### 2.3. Total Serum IgE

Total serum IgE at Week 6 was higher in OVA treated groups (the OVA group: 755 ± 231, the OVA + PM group: 558 ± 131 ng/mL), compared to control (162 ± 41.5 ng/mL) (*p* < 0.01) and the PM group (174 ± 94 ng/mL) (*p* < 0.01). Total serum IgE was similar between the OVA group and the OVA + PM group, and between the PM group and control (*p* > 0.05) (Figure 2D).

### 2.4. Gene Transcription Profile

**Figure 3:**
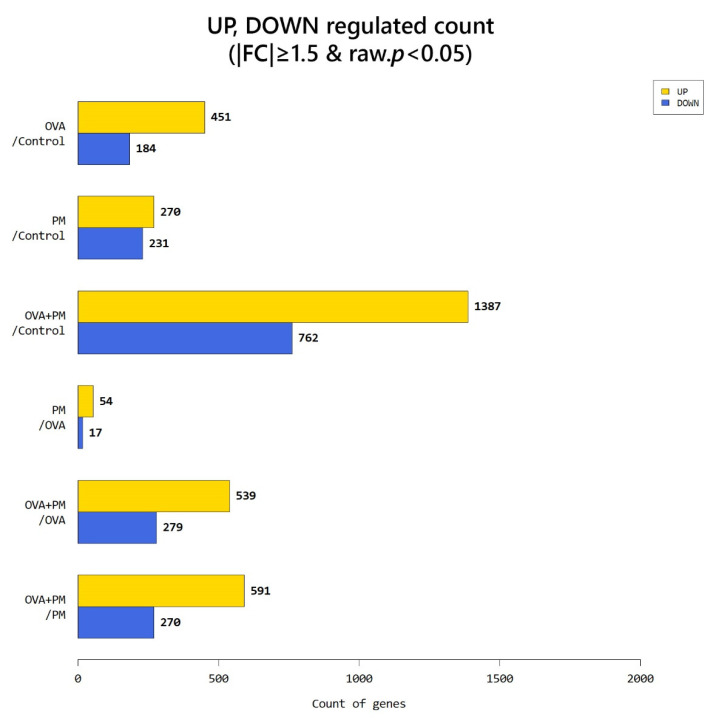
An overview of the differentially expressed genes (DEGs).

**Figure 4:**
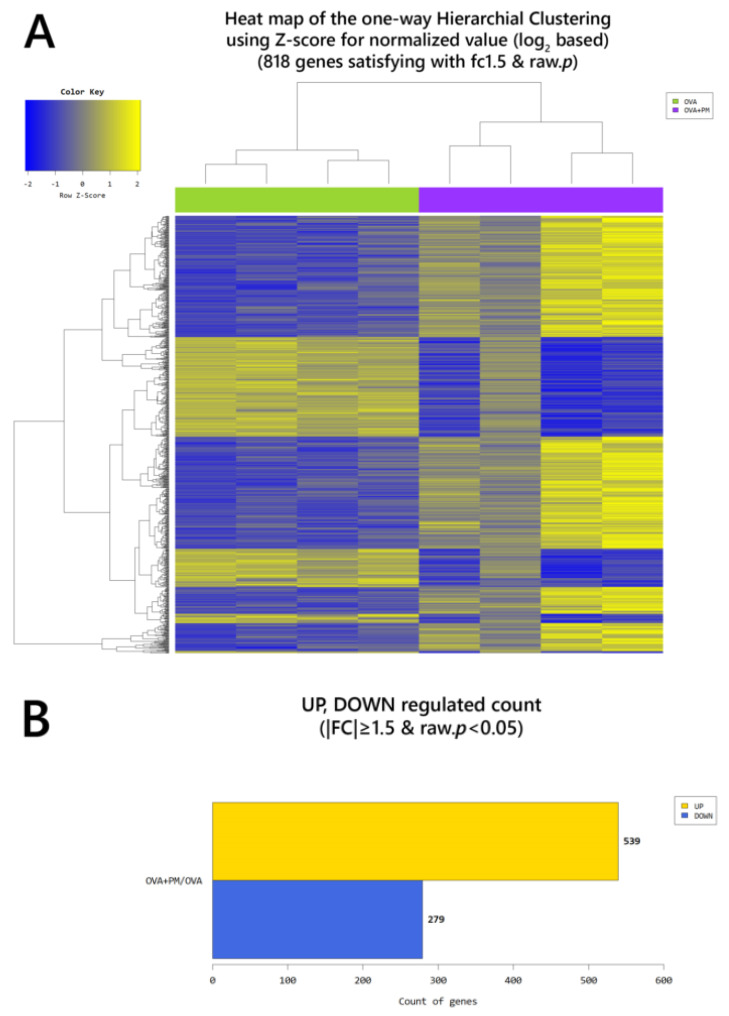

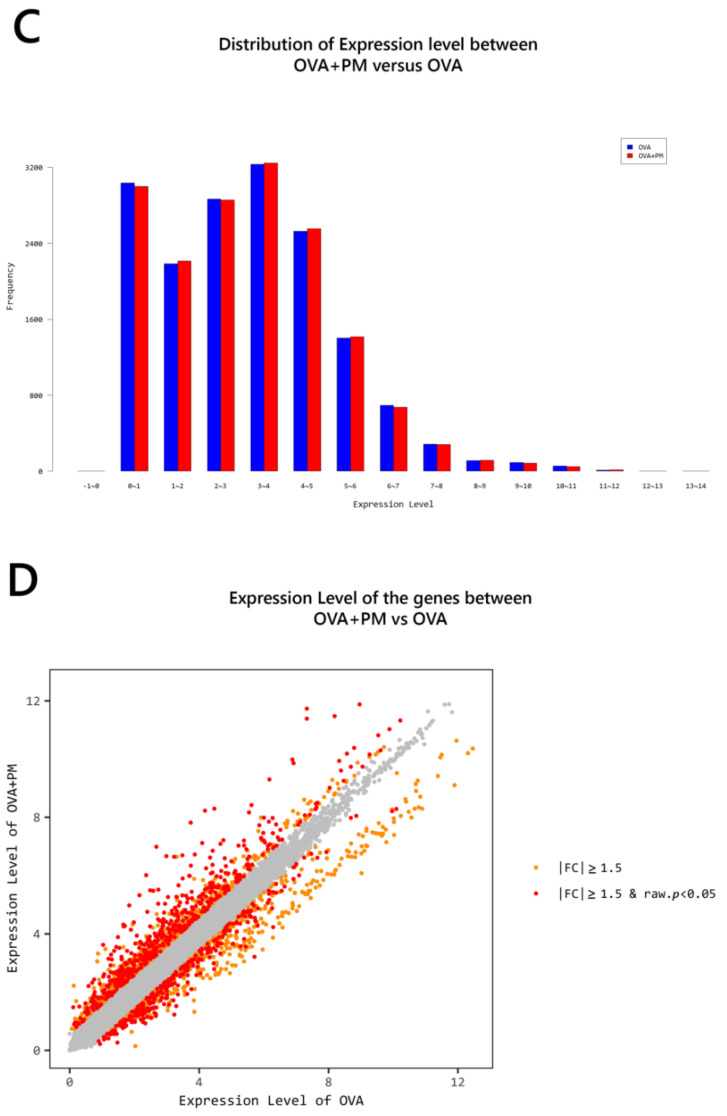
(**A**) Heat map of the one-way hierarchical clustering (OVA + PM vs. OVA). (**B**) Distribution of gene expression level between the OVA + PM and the OVA group. (**C**) Scatter plot of gene expression level. (**D**) Significant gene count by fold change and *p*-value. *n* = 4 in each group (OVA + PM, OVA). Only those genes exhibiting log_2_ fold change (FC) ≥ 1.5 and *p* < 0.05 were considered differentially expressed genes. For the DEG (differentially expressed gene) set, hierarchical clustering analysis was done using complete linkage and Euclidean distance as a measure of similarity.

**Figure 5:**
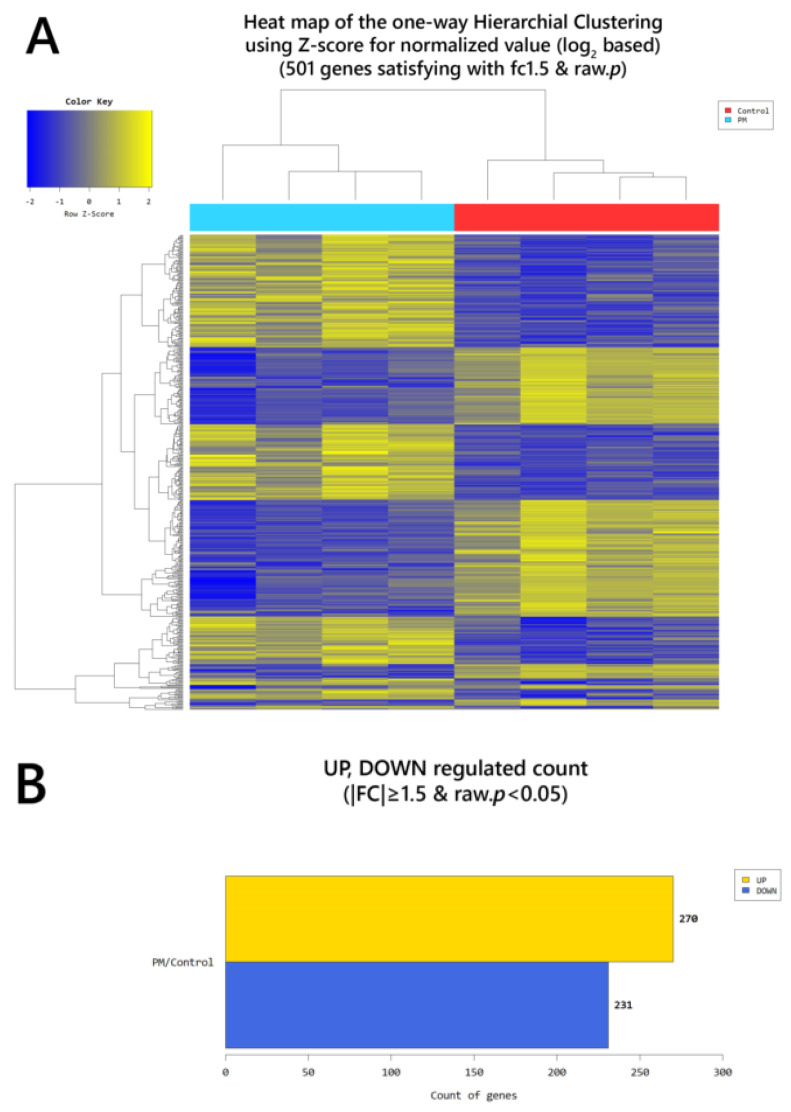

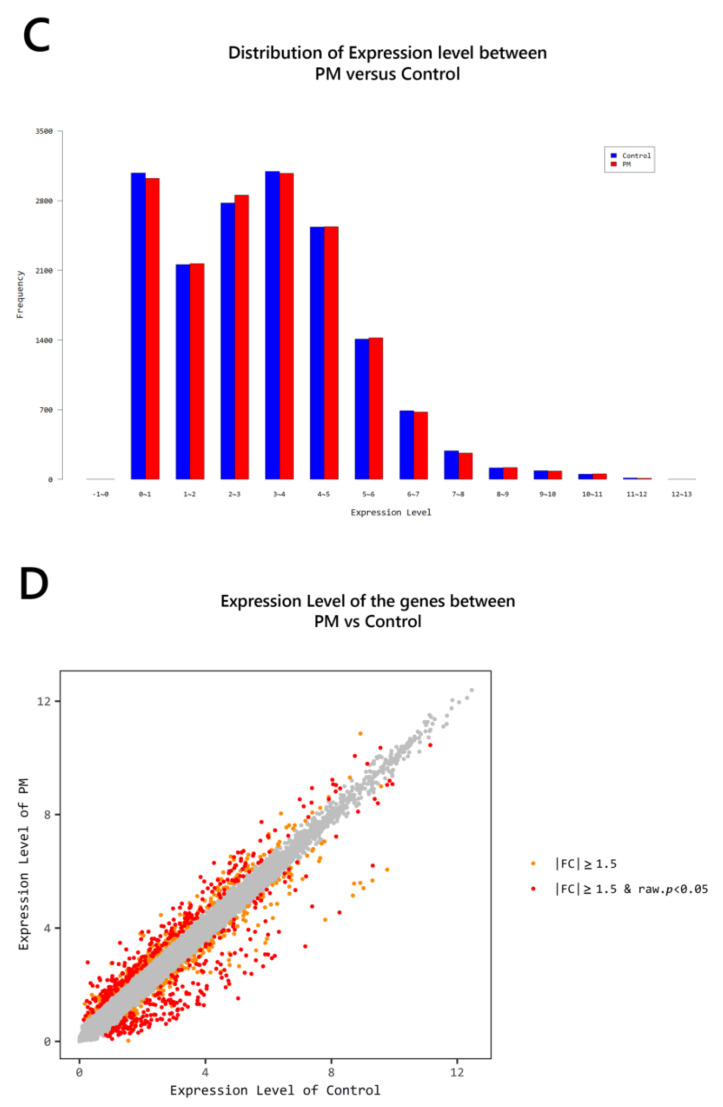
(**A**) Heat map of the one-way hierarchical clustering (PM vs. control). (**B**) Distribution of gene expression level between the PM and the control group. (**C**) Scatter plot of gene expression level. (**D**) Significant gene count by fold change and *p*-value. *n* = 4 in each group (PM, control). Only those genes exhibiting log_2_ fold change (FC) ≥ 1.5 and *p* < 0.05 were considered differentially expressed genes.

According to RNA-Seq analysis, a total of 635 genes were found to be differentially expressed by OVA exposure (greater than 1.5-log_2_ folds up and down and a raw *p*-value < 0.05). Among the 635 genes, 451 genes were upregulated, and 184 downregulated. In the PM exposed group, a total of 501 genes were differentially expressed (270 upregulated and 231 downregulated). With OVA + PM_10_ application, the differentially expressed gene (DEG) count was 2149 (1387 upregulated and 762 downregulated). Between the OVA + PM and the OVA group, the number of DEGs was 818 (539 upregulated and 279 genes downregulated). In comparing OVA + PM_10_ application to PM_10_ alone, a total of 861 DEGs were found (591 upregulated and 270 downregulated). Between the PM and the OVA group, 71 genes were differentially expressed (54 genes upregulated and 17 downregulated) (Figure 3). The heat map and volcano plots comparing the OVA + PM group and the OVA group and the PM group vs. control are presented in Figure 4 and Figure 5, respectively.

The top 50 significantly up-regulated genes ranked according to the fold change are presented in Supplementary Table S1. Among them, *SPRR2D*, *S100A9*, *STFA3*, *CHIL1*, *DBP*, *IL1b*, *SPRR2A1*, *LCE1H*, *SPRR2B*, *LCE1G* (Group PM vs. control), *RNASE2A*, *S100A9*, *SPRR2D*, *THRSP*, *SPRR2A1*, *S100A8*, *SERPINB3A*, *SPRR2B*, *C1QTNF3*, *CXCL1* (Group OVA vs. control), *S100A9*, *SPRR2D*, *SPRR2B*, *S100A8*, *SPRR2A3*, *SERPINB3A*, *STFA3*, *SPRR2A1*, *SPRR2E*, *BC100530* (Group OVA + PM vs. control), *S100A9*, *SPRR2B*, *SAA3*, *S100A8*, *SPRR2D*, *SPRR2A3*, *SERPINB3A*, *SPRR2E*, *GM5416*, *STFA3* (Group OVA + PM vs. Group OVA), *S100A9*, *S100A8*, *SAA3*, *SPRR2B*, *SPRR2D*, *SPRR2A3*, *SERPINB3A*, *SPRR2E*, *GM5416*, *SPRR2A1* (Group OVA + PM vs. Group PM), *NPY*, *FAM3B*, *GUCA2A*, *WFDC3*, *IL22RA2*, *UGT1A1*, *TESC*, *SERPINE2*, *CRABP1*, *PTGS1* (Group PM vs. Group OVA) were most significantly up-regulated (Table 1). The DEGs are also presented in Table 2 according to their function.

**Figure 6:**
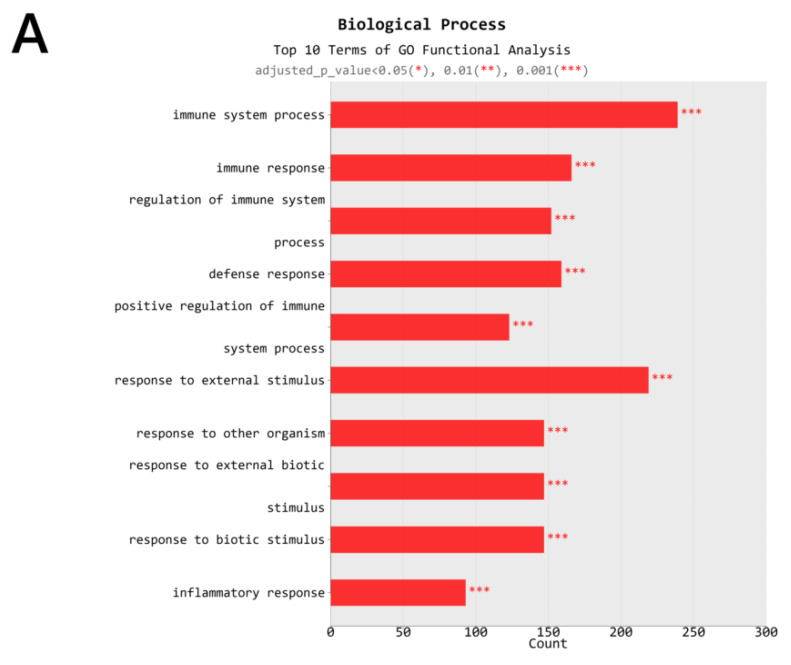

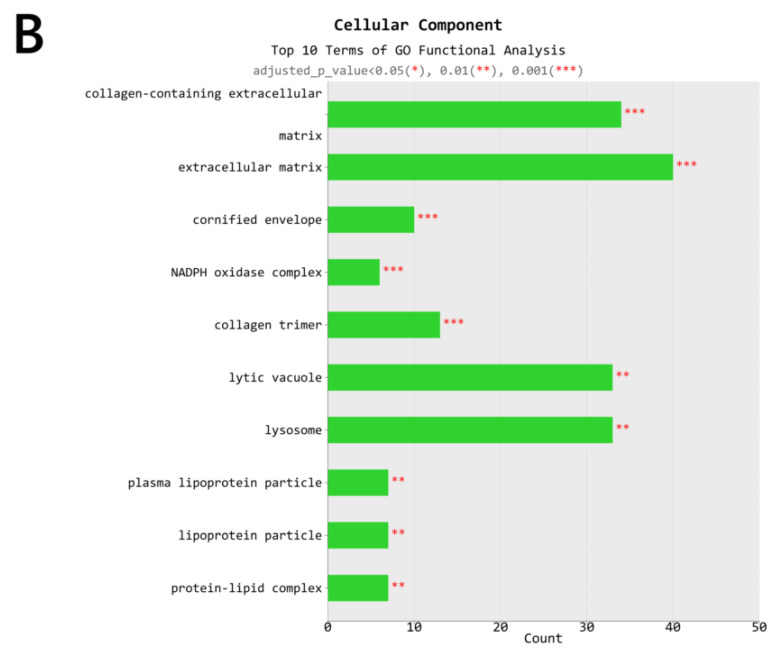

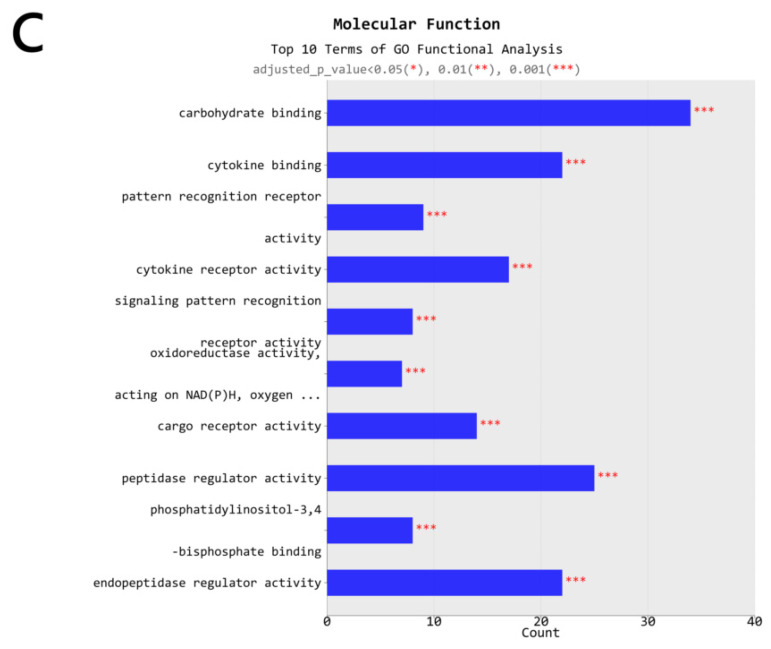
The major gene ontology (GO) terms and pathways of OVA + PM vs. OVA group. (**A**) Biologic process, (**B**) Cellular component, (**C**) molecular function.

The major gene ontology (GO) terms and pathways of OVA + PM vs. OVA group are shown in Figure 6. As for the biological process, the top 10 terms of GO functional analysis were immune system process, immune response, regulation of immune system process, defense response, positive regulation of immune system process, response to external stimulus, response to other organism, response to external biotic stimulus, response to biotic stimulus, and inflammatory response (Figure 6A). The cellular component included cornified envelope and the NADPH (nicotinamide adenine dinucleotide phosphate oxidase) complex (Figure 6B), and the molecular function included cytokine binding, oxidoreductase activity acting on NADPH, pattern recognition receptor activity and peptidase regulator activity (Figure 6C). KEGG analysis showed that up-regulated DEGs for both OVA + PM vs. OVA group and PM vs. control group were enriched in metabolic pathways (mmu01100), Ras signaling pathway (mmu04014), Rap1 signaling pathway (mmu04015), MAPK signaling pathway (mmu04010), Jak-STAT signaling pathway (mmu04630), NF-kappa B signaling pathway (mmu04064), TNF signaling pathway (mmu04668), HIF-1 signaling pathway (mmu04066), calcium signaling pathway (mmu04020), cytokine-cytokine receptor interaction (mmu04060), toll-like receptor signaling pathway (mmu04620), nodulation (NOD)-like receptor signaling pathway (mmu04621), c-type lectin receptor signaling pathway (mmu04625), Th17 cell differentiation (mmu04659), IL-17 signaling pathway (mmu04657), inflammatory mediator regulation of TRP channels (mmu04750), and pathways in cancer (mmu05200).

**Figure 7:**
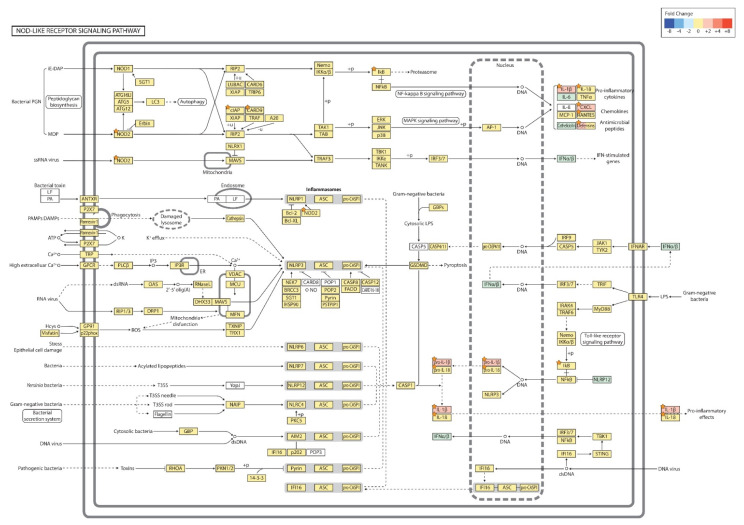
Nodulation (NOD)-like receptor signaling pathway (PM vs. control). The orange star signifies genes with a *p* < 0.05. +p: phosphorylation; +u: ubiquitination; −u: de-ubiquitination; - - ->: activation.

The NOD-like receptor signaling pathway is shown in Figure 7. Among the relevant genes, cutaneous PM_10_ exposure induced up-regulation of *NOD2* (log_2_ FC: 1.505669), *NFKBIA* (log_2_ FC: 1.856415), *CARD9* (log_2_ FC: 1.655884), *IL1B* (log_2_ FC: 3.375845), *IL18* (log_2_ FC: 1.545413), *CXCL1* (log_2_ FC: 2.034245), *CCL5* (log_2_ FC: 1.608780), *DEFB14* (log_2_ FC: 2.276974), and *BIRC3* (log_2_ FC: 1.60949) (*p*-value < 0.05).

**Figure 8:**
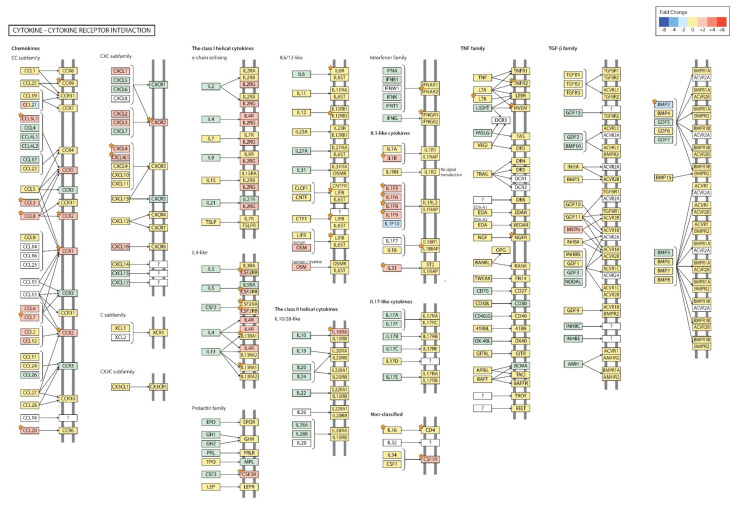
The cytokine-cytokine receptor interaction pathway (OVA + PM vs. OVA). The orange star signifies genes with a *p* < 0.05; ?: unknown.

The cytokine-cytokine receptor interaction pathway of OVA + PM group showed up-regulation of *IL1B* (log_2_ FC: 7.414423), *CCL8* (log_2_ FC: 3.054375), *CCL20* (log_2_ FC: 3.529423), *CCR1* (log_2_ FC: 3.623166), *CCR2* (log_2_ FC: 2.525612), *CCR9* (log_2_ FC: 1.530475), *CXCR2* (log_2_ FC: 3.934086), *CSFLR* (log_2_ FC: 2.709420), *Il10RA* (log_2_ FC: 2.133591), *Il1F5* (log_2_ FC: 2.587269), *Il1F6* (log_2_ FC: 3.870511), *Il1F8* (log_2_ FC: 2.818568), *Il1F9* (log_2_ FC: 4.090919), *PF4* (log_2_ FC: 3.212812), *Il33* (log_2_ FC: 3.116752), *Il18RAP* (log_2_ FC: 1.976295), *Il12RB2* (log_2_ FC: 1.938648), *Il13RA1* (log_2_ FC: 1.786709), *LTB* (log_2_ FC: 1.560564), *TNFRSF1B* (log_2_ FC: 1.784543), *TNFRSF14* (log_2_ FC: 1.711990), *IFNAR2* (log_2_ FC: 1.628548), *CD4* (log_2_ FC: 1.621931), *IL6RA* (log_2_ FC: 1.616884) and the downregulation of *BMP2* (log_2_ FC: −2.171518) and *NGFR* (log_2_ FC: −1.737021) compared to the OVA group (*p*-value < 0.05) (Figure 8).

## 3. Discussion

This study explored how exposure to PM_10_ modulates the development and exacerbation of AD using OVA-treated BALB/c mice. The endpoints of this study included: (1) the extent of clinical and histological skin inflammation including hallmarks of allergic inflammation; and (2) the expression of various genes involved in the skin barrier and immune response to gain insight into the PM modulation of AD.

Our OVA exposed mice successfully captured the characteristics of AD (i.e., increase in serum IgE, mast cell infiltration in the dermis, elevated gene expression of *CHIL1* (related to Th2 response), *FCER1A* (Fc fragment of IgE receptor 1a), *IL-33* (an epithelial cell-derived cytokine that promotes Th2 cytokine responses), and *RNASE2A* (important for eosinophil recruitment and function)). Key AD genes, including the Th2 and Th22 cytokines (*IL-4*, *IL-13*, *IL-22*) are usually present at less than detection level on microarrays, requiring real-time PCR (RT-PCR) [21,22,23]. This was also the case with our samples—although absent from microarray, we were able to detect IL-13 in the OVA treated groups through real-time PCR (RT-PCR) (data not shown).

No single murine model fully captures all aspects of the AD profile. Ewald et al. [24] have recently compared the transcriptomic profiles of common AD-like murine models and identified that the OVA-challenged model has significant overlap with genes upregulated in human AD, but does not capture the downregulated signature of human AD. Accordingly, we tried to focus on the upregulated genes in our study. The DEGs of our OVA exposed mice and those of Ewald et al.’s [24] OVA-challenged model were highly similar, which confirmed the reliability of our AD mouse model.

PM_10_ displayed adjuvant-like effects, enhancing skin inflammation/barrier damage upon OVA challenge (i.e., enhanced skin severity scores, TEWL, epidermal thickness, and increased expression of skin barrier genes (epidermal differentiation complex: *KRT1*, *6b*, *16*; *LCE3A*, *3B*, *3E*, *3F*; *S100A8*, *A9*; *SPRR2A1*, *2A3*, *2B*, *2D*, *2E*, *2I*; protease: *MMP3*; *SERPINB3A*, *3B*; *STFA1*, *3*; *BC100530*; *KLK8*, *9*, *13*; antimicrobial response: *DEFB14*), and pro-inflammatory genes (*IL-1B*, *TNF1IP2*). The expression of allergy genes (*IL-13RA1*, *IL-33*, *FCERIG*, *CHIL1*) was also enhanced in the OVA + PM group when compared to the OVA group indicating the possible exacerbation of AD.

We were also intrigued to see if PM_10_ affects intact skin. In a prior study, Jin et al. [25] have detected PM inside hair follicles in both intact and barrier-disrupted skin. Additionally, repeated PM application was shown to induce epidermal thickening and dermal inflammation with neutrophil infiltration. Although we failed to detect PM in the appendageal structures/dermis of our skin sections, our findings were similar with that of Jin et al. [25], where enhanced skin severity scores, TEWL, epidermal thickness, and increased expression of skin barrier genes (epidermal differentiation complex: *KRT1*; *LCE1F*, *1G*, *1H*; *LCE3E*, *3F*; *S100A8*, *A9*; *SPRR2A1*, *2A3*, *2B*, *2D*, *2E*, *2I*; protease: *SERPINB3A*, *3B*; *STFA1*, *3*; *BC100530*; *KLK8*, *9*, *13*; antimicrobial response: *DEFB14*; other: *2610528A11RIK*) and pro-inflammatory genes (*IL-1B*, *CXCL1*) were noted. The increase in mast cell number, heightened expression of an allergy-related gene (*CHIL1*), and detection of *IL-13* through RT-PCR (data not shown) suggest that AD can perhaps develop following PM_10_ exposure alone.

The main cause of PM-inflicted skin damage has been identified as polycyclic aromatic hydrocarbons (PAHs), the main organic constituent of PM [15,26,27]. PAHs exert their biological effect via binding to the ligand-activated transcription factor aryl hydrocarbon receptor (AHR), which is widely expressed on skin cells [28]. AHR is a major sensor of environmental signals, but at the same time, AHR ligands are abundant in the skin from exogenous or endogenous sources [28].

The quality and duration of AHR activation by various ligands directs the level and spectrum of the genes which are induced, and are thus pivotal in the outcome, including a “toxic” outcome [29,30]. Three important groups of genes are targeted by AHR [29]. First, a battery of genes encoding detoxifying enzymes (xenobiotic metabolizing enzymes, *XMEs*), such as the cytochrome P450 (CYP) gene *CYP1A1* (Phase I XME) and Phase II enzymes (NADPH dehydrogenase quinone 1, *NQO1*; glutathione S-transferases, *GSTA2*; uridine 5-diphospho-glucuronosyltransferases; *UGT1A1*, *UGT1A6*, *UGT1A7*) [31,32,33]; second, genes related to epidermal differentiation and skin barrier integrity; and finally, genes related to immunity.

The AHR battery genes are noteworthy in that we have found evidence of aberrant AHR activation with our model (OVA group, PM group, OVA + PM group) based on elevated gene expression levels of XMEs. Xenobiotic small chemicals have strong affinity to AHR and cause persistent activation of the receptor [28]. The pathogenic implication of AHR and its gene polymorphism in AD remain elusive but it has been suggested that most AHRs lack physiological ligands in the Th2-prone milieu in AD [31,34].

Transgenic mice expressing constitutively active form of AHR (AHR-CA) [35] (surmised to be equivalent to PAH-liganded AHR) have shown a gene profile with an increase in structural protein genes (*KRT1*, *6B*, *16*), protease genes (i.e., *MMP*), interleukins/chemokine genes (i.e., *IL-1B*, *CXCL1*, *CCL8*), Fc receptor genes (*FCERIG*), and antimicrobial peptide genes (i.e., *DEFB*) reproduced in our mouse models (PM group, OVA + PM group), which indicates the role of the PAH-liganded AHR in PM induced skin barrier dysfunction/immune deviation.

The ligation of AHR by xenobiotic small chemicals (i.e., PAH, dioxin) was reported to preferentially affect the differentiation and propagation of Th 17 cells [31,36,37], as seen in our PM exposed mouse models (enrichment of upregulated DEGs in the Th17 cell differentiation (mmu04659), and IL-17 signaling pathway (mmu04657)), which too suggests that the PAH-AHR axis underlies the allergic response to PM.

PAH itself has also been suggested to provoke inflammation as a primary irritant or allergen [35,38,39,40]. Other lines of evidence suggest that reactive oxygen species (ROS) generated by oxygenated PAHs enhance the allergic response [41,42]. PAHs have also been shown to stimulate an increase in the DNA-binding activity of NF-kB [43], which, in turn, induces cytokine gene expression provoking the allergic response. To note, the NF-kappa B signaling pathway (mmu04064) was found to be enriched with upregulated DEGs in our OVA + PM and PM group.

In summary, we demonstrate that PM exacerbates AD when exposure occurs during simultaneous allergen sensitization/elicitation. The enhancement of the allergic immune response by PM is characterized by increased mast cells in the dermis, elevated serum IgE level, upregulated expression of the skin barrier genes (epidermal differentiation complex; protease; antimicrobial response), pro-inflammatory genes, and allergy genes (microarray: *IL-13RA1*, *IL-33*, *FCERIG*, *CHIL1*; RT-PCR: *IL-13*; KEGG analysis: Th17 cell differentiation, IL-17 signaling pathway). PM-mediated toxicity may be the result of PAHs modulating immunity and the epidermal barrier via the AHR.

Since PM is also able to initiate AD in intact skin, further work is needed to investigate if PM enhances the antigen-presenting capabilities of dendritic cells, and if this translates to enhanced B and T cell adaptive responses, as well as the critical role of the AHR in these processes. Our identification of the molecular mechanisms through which PM mediates its toxicological effects and enhances immune-mediated inflammation and barrier damage sheds light on the sharp rise of AD in the past decades.

In conclusion, we provide evidence on the importance of public awareness in PM-associated skin inflammation. Vigilant attention and preventive measures should be paid to all individuals, especially to those with AD.

## 4. Materials and Methods

### 4.1. Particulate Matter

PM_10_ was collected in 2005 from an air intake filtration system of a major exhibition center in Prague, Czech Republic (NIST, SRM 2787). PM suspension was freshly prepared by resuspending PM particles in phosphate-buffered saline (PBS) at a concentration of 2.5 mg/mL, and vortexing for 30 min at maximum speed.

### 4.2. Animals

Four-week-old female BALB/c mice were procured from Orient Bio Inc., Sungnam, Korea. Animals were housed in specific pathogen-free (SPF) environment, exposed to a 12-h light/dark cycle, and were provided with autoclaved water and food ad libitum. The mice were randomly divided into 4 groups (control group, OVA group, PM group, OVA + PM group; *n* = 6). After a week of acclimatization, the back of the mice was shaved with an electric clipper (day 0) and was kept hair-free with hair removal cream (Veet) and tape strips (Nad’s body wax strip) twice weekly for the entire study period. The study protocol was approved by the Institutional Animal Care and Use Committees (IACUCs) of the College of Medicine, The Catholic University of Korea (2019-0207-03, 1 August 2019).

### 4.3. Sensitization and Challenge

**Figure 9:**
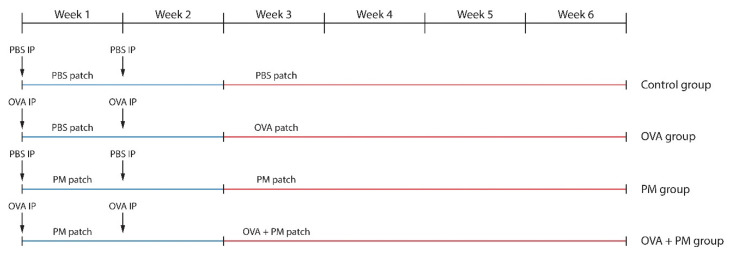
Experimental protocol for assessing the effects of OVA (IP + cutaneous) and PM_10_ (cutaneous) exposure in BALB/c mice.

The schematic experimental procedure is described in Figure 9. The mice in the OVA group and OVA + PM group were intraperitoneally (IP) injected with 20 μg chicken egg ovalbumin (OVA) (A5503-1G, Sigma-Aldrich, St. Louis, MO, USA) and 2 mg of aluminum hydroxide (769460-100G, Sigma-Aldrich, St. Louis, MO, USA) in 200 μL of PBS on days 0, and 7 using a modified protocol [44,45]. Those in the control group and the PM group were IP injected with an equal volume of PBS on the same date. From day 0, a PM patch (250 μg/cm^2^ of PM_10_ applied on a nonwoven 2 × 2 cm^2^ polyethylene sheet (Scotch Brite^TM^, 3M, St. Paul, MN, USA) and fixed with a transparent adhesive film dressing (Tegaderm^TM^, 3M, St. Paul, MN, USA) was applied daily to the backs of the PM group (until Week 6) and the OVA + PM group (until Week 2) mice. A PBS patch (400 μL of PBS applied on a 2 × 2 cm^2^ nonwoven polyethylene sheet and fixed with a transparent adhesive film dressing) was employed in the same manner in the control group (until Week 6) and the OVA group (until Week 2). Seven days after the final IP injection, mice in the OVA group and the OVA + PM group were challenged with OVA (400 μg of OVA dissolved in 400 μL of PBS applied on a 2 × 2 cm^2^ nonwoven polyethylene sheet and fixed with a transparent adhesive film dressing) and OVA + PM (400 μg of OVA + 250 μg/cm^2^ of PM_10_ in 400 μL of PBS applied on a nonwoven 2 × 2 cm^2^ polyethylene sheet and fixed with a transparent adhesive film dressing patches respectively, until the end of the study (Week 6).

### 4.4. Assessment of Clinical Parameters

Clinical assessments were made twice a week for the entire study period. The trans-epidermal water loss (TEWL) was assessed on the dorsal skin of the BALB/c mice using the VapoMeter (Delfin Technologies, Kuopio, Finland). A modified scoring atopic dermatitis (SCORAD) (defined as the sum of individual scores for each of the following 4 signs and symptoms: erythema, edema, excoriation, and dryness. Each item was scored as 0 (none), 1 (mild), 2 (moderate), and 3 (severe), as previously described) was used to measure the clinical severity. Scoring was performed by 2 assessors masked to the study purpose and hypothesis. They were not involved in treatment administration or assignment.

### 4.5. Histopathology

The mice were sacrificed in Week 6. The dorsal skin samples were fixed in 10% vol. phosphate-buffered formalin solution, embedded in paraffin, and sectioned at 4 μm. The tissue sections were stained with hematoxylin and eosin (H&E) for microscopic examination. For identification of mast cells, skin sections were stained with toluidine blue. The mast cells were counted in 5 randomly chosen visuals fields at ×0400 magnification. The evaluation was performed at a central laboratory, where slides were made available for a central reading by an assessor masked to the experiment.

### 4.6. Enzyme-Linked Immunosorbent Assay (ELISA)

Blood was collected from the retroorbital plexus using heparinized glass capillary tubes at the end of the experiment (Week 6). Serum samples obtained by centrifugation (3000× *g* for 4 min at 4 °C) were stored at −80 °C until use. Concentration of total IgE serum was determined using the mouse IgE ELISA kit (Shibayagi Co. Ltd., Gunma, Japan), according to the manufacturer’s instruction. Laboratory evaluations were performed at a central laboratory.

### 4.7. mRNA-Seq

Total RNA concentration was calculated by Quant-IT RiboGreen (R11490, Invitrogen, Carlsbad, CA, USA). To assess the integrity of the total RNA, samples were run on the TapeStation RNA screentape (#5067-5576, Agilent, Santa Clara, CA, USA). Only high-quality RNA preparations, with RIN greater than 7.0, were used for RNA library construction.

cDNA libraries were constructed with the TruSeq RNA library kit (RS-122-2101, Illumina Inc., San Diego, CA, USA) where 1 μg of RNA was used per sample. RNA was polyA-selected, fragmented, reverse transcribed and sequenced with Illumina HiSeq4000 (San Diego, CA, USA). Libraries were quantified with the qPCR-based KAPA Library Quantification Kit (KK4854) and qualified with an Agilent Technologies 2100 Bioanalyzer (Santa Clara, CA, USA). 

The raw reads were preprocessed and then aligned to *Mus musculus* (*mm*10) with HISAT v2.2.0 (http://ccb.jhu.edu/software/hisat2/) [46]. HISAT employs two kinds of indexes and creates spliced alignments faster than BWA and Bowtie. Downloads of the reference genome sequence and annotation data are available from http://genome.uscs.edu. StringTie v1.3.4d (http://ccb.jhu.edu/software/stringtie/) [47,48] was used to build aligned reads into transcripts and calculate fragments per kilobase of exon per million fragments mapped (FPKM) values. Standardized FPKM values were utilized to compare gene’s expression levels. Sixteen samples (control, OVA, PM, OVA + PM groups; 4 samples per group) were examined in total.

### 4.8. Statistical Analysis

All data are expressed as the mean ± SD. One-way analysis variance (ANOVA,) followed by the Tukey multiple comparison test, was used to assess differences in the measurements between multiple groups. Statistical analyses were performed using Graph Pad Prism 4.0 (San Diego, CA, USA). A *p*-value of less than 0.05 was considered statistically significant.

Statistical analysis was carried out to find DEGs. Transcripts with zeroed FPKM values were eliminated. Filtered data were log_2_-transformed and quantile normalized. Statistical significance of the DEG data was verified with independent *t*-test and fold change with a null (no difference) hypothesis. The false discovery rate (FDR) was corrected with the Benjamini-Hochberg algorithm. Hierarchical clustering analysis was performed employing Euclidean distance and complete linkage. Gene-enrichment and functional annotation analysis and pathway analysis for significant gene list were carried out according to gProfiler (http://biit.cs.ut.ee/gprofiler/orth) and KEGG pathway (http://www.genome.jp/kegg/pathway.html).

## Figures and Tables

**Table 1:** Top 10 significantly upregulated genes.

	PM vs. Control	OVA vs. Control	OVA + PM vs. Control	OVA + PM vs. OVA	OVA + PM vs. PM	PM vs. OVA
**1**	*SPRR2D*	*RNASE2A*	*S100A9*	*S100A9*	*S100A9*	*NPY*
Log_2_FC	4.537781	15.353418	175.102606	21.032206	43.232510	2.912855
**2**	*S100A9*	*S100A9*	*SPRR2D*	*SPRR2B*	*S100A8*	*FAM3B*
Log_2_FC	4.050253	8.325451	122.348283	19.765663	36.247161	2.237210
**3**	*STFA3*	*SPRR2D*	*SPRR2B*	*SAA3*	*SAA3*	*GUCA2A*
Log_2_FC	3.884888	7.414729	99.190350	16.871093	33.226551	2.082410
**4**	*CHIL1*	*THRSP*	*S100A8*	*S100A8*	*SPRR2B*	*WFDC3*
Log_2_FC	3.852430	6.074408	86.466724	16.615182	30.495545	2.057602
**5**	*DBP*	*SPRR2A1*	*SPRR2A3*	*SPRR2D*	*SPRR2D*	*IL22RA2*
Log_2_FC	3.637145	5.429848	68.747026	16.500708	26.962138	1.771707
**6**	*IL1B*	*S100A8*	*SEPINB3A*	*SPRR2A3*	*SPRR2A3*	*UGT1A1*
Log_2_FC	3.375845	5.204079	65.385001	14.139278	21.554361	1.765702
**7**	*SPRR2A1*	*SERPINB3A*	*STFA3*	*SERPINB3A*	*SERPINB3A*	*TESC*
Log_2_FC	3.293317	5.127475	51.600623	12.751891	21.542993	1.728145
**8**	*LCE1H*	*SPRR2B*	*SPRR2A1*	*SPRR2E*	*SPRR2E*	*SERPINE2*
Log_2_FC	3.272727	5.018316	50.181213	12.506733	16.809473	1.705879
**9**	*SPRR2B*	*C1QTNF3*	*SPRR2E*	*GM5416*	*GM5416*	*CRABP1*
Log_2_FC	3.252618	4.536723	43.315581	10.651026	16.545459	1.702428
**10**	*2610528A11RIK*	*CXCL1*	*BC100530*	*STFA3*	*SPRR2A1*	*PTGS1*
Log_2_FC	3.202117	3.865055	29.494805	9.717934	15.237285	1.689424

**Table 2:** DEGs according to their function.

Genes	PM vs. Control	OVA vs. Control	OVA + PM vs. Control	OVA + PM vs. OVA	OVA + PM vs. PM	PM vs. OVA
**Xenobiotic Metabolizing Enzyme**						
*CYP1A1*	2.364800					
*UGT1A1*	1.968411					1.765702
*UGT1A7C*		1.563135	4.020299	2.571945	2.447144	
**Immune Response**						
*IL1B*	3.375845	2.144279	15.898589	7.414423	4.709514	
*IL1F6*	2.459084	2.677326	10.362620	3.870511	4.214016	
*IL1F8*	1.682234	1.930127	5.440192	2.818568	3.220411	
*IL1F9*	2.309176	1.647147	6.738345	4.090919		
*IL-13ra1*			2.339857	1.786709	1.753326	
*IL-13ra2*			2.445235		1.997591	
*IL-33*		1.652810	5.151400	3.116752	3.571956	
*CXCL1*	2.034245	3.865055	15.266021		7.504513	
*CCL2*		2.414083	3.966503			
*CCL7*		3.176686	6.829295	2.149817	3.330636	
*CCL8*		3.262495	9.964881	3.054375	7.572985	
*CCR1*		1.977892	7.166230	3.623166	5.588996	
*CXCR2*			5.409111	3.934086	4.409069	
*TNFAIP2*			2.981176	2.211034	2.536282	
*TNFAIP6*		3.665933	3.788700			
*FCER1A*		3.066804	5.700756		3.742706	
*FCER1G*		1.866897	4.525466	2.424057	3.528595	
*CHIL1*	3.852430	3.413080	23.799435	6.973008	6.177772	
*RNASE2A*		15.353418	17.480094		5.250776	−4.611952
**Skin Barrier**						
**Epidermal Differentiation Complex**						
*KRT1*	2.012479		5.013296	3.007899	2.4911105	
*KRT6b*			9.746359	7.671900	10.906282	
*KRT16*			14.027059	8.550596	9.550772	
*LCE1F*	2.848601	1.856882	2.793710			
*LCE1G*	3.202117	2.625508	3.709355			
*LCE1H*	3.272727	2.649690	3.867855			
*LCE3A*		1.985872	11.751650	5.917627	7.581050	
*LCE3B*			12.008217	6.312606	9.559686	
*LCE3E*	1.877260		9.465151	5.314134	5.042002	
*LCE3F*	1.999833		11.052984	4.905179	5.526953	
*S100A8*	2.385476	5.204079	86.466724	16.615182	36.247161	
*S100A9*	4.050253	8.325451	175.102606	21.032206	43.232510	
*SPRR2A1*	3.293317	5.429848	50.181213	9.241734	15.237285	
*SPRR2A3*	3.189472		68.747026	14.139278	21.554361	
*SPRR2B*	3.252618	5.018316	99.190350	19.765663	30.495545	
*SPRR2D*	4.537781	7.414729	122.348283	16.500708	26.962138	
*SPRR2E*	2.576855	3.463381	43.315581	12.506733	16.809473	
*SPRR2I*	1.924438	2.981781	26.165916	8.775266	13.596652	
*FLG*			−1.749558		−2.481263	
**Protease**						
*MMP3*		2.031330	10.906441	5.369114	6.745412	
*SERPINB3A*	3.035094	5.127475	65.385001	12.751891	21.542993	
*SERPINB3B*	2.497089	2.768278	17.026990	6.150751	6.818736	
*STFA1*	2.008981		17.513492	6.855981	8.717599	
*STFA3*	3.884888		51.600623	9.717934	13.282395	
*BC100530*	2.912126		29.494805	7.555283	10.128274	
*KLK6*			3.837728			
*KLK8*	2.456366	1.996790	6.233008	3.121514		
*KLK9*	2.747518	1.846616	9.784243	5.298470		
*KLK13*	2.201600	2.251518	13.571149	6.027555	6.164222	
**Antimicrobial Response**						
*DEFB6*	1.852542	1.683457	3.312694			
*DEFB14*	2.278557		5.154124	2.628721		
**Other**						
*2610528A11RIK*	3.128210	3.748969	27.119970	7.233981	8.669484	

## Supplementary Materials

The following are available online at https://www.mdpi.com/1422-0067/21/17/6079/s1.

## Abbreviations

AD	Atopic dermatitis
PM	Particulate matter
OVA	Ovalbumin
TEWL	Trans-epidermal water loss
PAHs	Polyaromatic hydrocarbons
ROS	Reactive oxygen species
SCORAD	Scoring atopic dermatitis
H&E	Hematoxylin and eosin
DEG	Differentially expressed genes
SPRR	Small proline rich proteins
STFA	Stefin A
CHIL	Chitinase-like
DBP	D site-binding protein
LCE	Late cornified envelope
RNASE	Ribonuclease
THRSP	Thyroid hormone responsive
SERPIN	Serine proteinase inhibitor
C1QTNF	C1q and tumor necrosis factor
CXCL	C-X-C motif chemokine ligand
SAA	Serum amyloid A
NPY	Neuropeptide Y
FAM3B	Family with sequence similarity 3, member B
GUCA	Guanylate cyclase activator
WFDC	WAP four-disulfide core domain
UGT	UDP glucuronosyltransferase
TESC	Tescalcin
CRABP	Cellular retinoic acid binding protein
GO	Gene ontology
NADPH	Nicotinamide adenine dinucleotide phosphate
KEGG	Kyoto encyclopedia of genes and genomes
RAP	Ras-related protein
MAPK	Mitogen-activated protein kinase
JAK-STAT	Janus kinases-signal transducer and activator of transcription
NF-kB	Nuclear factor-kappa B
TNF	Tumor necrosis factor
HIF	Hypoxia-inducible factor
NOD	Nodulation
NFKBIA	NF-kappa-B inhibitor alpha
CARD	Caspase activation and recruitment domains
CCL	CC chemokine ligand
DEFB	Defensin beta
BIRC3	Baculovial IAP repeat containing 3
CCR	C-C chemokine receptor
CXCR	C-X-C chemokine receptor
CSF1R	Colony stimulation factor-1 receptor
PF4	Platelet factor 4
LTB	Lymphotoxin-beta
TNFRSF	Tumor necrosis factor receptor superfamily
IFNAR2	Interferon alpha and beta receptor subunit 2
BMP	Bone morphogenetic protein
NGFR	Nerve growth factor receptor
FCER	Fc fragment of IgE receptor
KRT	Keratin
KLK	Kallikreins
AHR	Aryl hydrocarbon receptor
XME	Xenobiotic metabolizing enzyme
CYP	Cytochrome P450
NQO	NADPH quinone oxidoreductase
GSTA	Glutathione S-transferase
UGT	UDP-glucuronosyltransferase
IP	Intraperitoneal
PBS	Phosphate-buffered saline

## References

[1] Hendricks A.J., Eichenfield L.F., Shi V.Y. (2020). The impact of airborne pollution on atopic dermatitis: A literature review. Br. J. Dermatol..

[2] Greco S.L., MacIntyre E., Young S., Warden H., Drudge C., Kim J., Candido E., Demers P., Copes R. (2020). An approach to estimating the environmental burden of cancer from known and probable carcinogens: Application to Ontario, Canada. BMC Public Health.

[3] Mueller W., Loh M., Vardoulakis S., Johnston H.J., Steinle S., Precha N., Kliengchuay W., Tantrakarnapa K., Cherrie J.W. (2020). Ambient particulate matter and biomass burning: An ecological time series study of respiratory and cardiovascular hospital visits in northern Thailand. Environ. Health.

[4] Moelling K., Broecker F. (2020). Air Microbiome and Pollution: Composition and Potential Effects on Human Health, Including SARS Coronavirus Infection. J. Environ. Public Health.

[5] Kim K.E., Cho D., Park H.J. (2016). Air pollution and skin diseases: Adverse effects of airborne particulate matter on various skin diseases. Life Sci..

[6] Park S.Y., Byun E.J., Lee J.D., Kim S., Kim H.S. (2018). Air Pollution, Autophagy, and Skin Aging: Impact of Particulate Matter (PM(10)) on Human Dermal Fibroblasts. Int. J. Mol. Sci..

[7] Araviiskaia E., Berardesca E., Bieber T., Gontijo G., Sanchez Viera M., Marrot L., Chuberre B., Dreno B. (2019). The impact of airborne pollution on skin. J. Eur. Acad. Dermatol. Venereol..

[8] Ahn K. (2014). The role of air pollutants in atopic dermatitis. J. Allergy Clin. Immunol..

[9] Pénard-Morand C., Raherison C., Charpin D., Kopferschmitt C., Lavaud F., Caillaud D., Annesi-Maesano I. (2010). Long-term exposure to close-proximity air pollution and asthma and allergies in urban children. Eur. Respir. J..

[10] Lee Y.L., Su H.J., Sheu H.M., Yu H.S., Guo Y.L. (2008). Traffic-related air pollution, climate, and prevalence of eczema in Taiwanese school children. J. Investig. Dermatol..

[11] Oh I., Lee J., Ahn K., Kim J., Kim Y.M., Sun Sim C., Kim Y. (2018). Association between particulate matter concentration and symptoms of atopic dermatitis in children living in an industrial urban area of South Korea. Environ. Res..

[12] Tang K.T., Ku K.C., Chen D.Y., Lin C.H., Tsuang B.J., Chen Y.H. (2017). Adult atopic dermatitis and exposure to air pollutants-a nationwide population-based study. Ann. Allergy Asthma Immunol..

[13] Kim Y.M., Kim J., Han Y., Jeon B.H., Cheong H.K., Ahn K. (2017). Short-term effects of weather and air pollution on atopic dermatitis symptoms in children: A panel study in Korea. PLoS ONE.

[14] Hassoun Y., James C., Bernstein D.I. (2019). The Effects of Air Pollution on the Development of Atopic Disease. Clin. Rev. Allergy Immunol..

[15] Hidaka T., Ogawa E., Kobayashi E.H., Suzuki T., Funayama R., Nagashima T., Fujimura T., Aiba S., Nakayama K., Okuyama R. (2017). The aryl hydrocarbon receptor AhR links atopic dermatitis and air pollution via induction of the neurotrophic factor artemin. Nat. Immunol..

[16] Guarnieri M., Balmes J.R. (2014). Outdoor air pollution and asthma. Lancet.

[17] Schantz M.M., Cleveland D., Heckert N.A., Kucklick J.R., Leigh S.D., Long S.E., Lynch J.M., Murphy K.E., Olfaz R., Pintar A.L. (2016). Development of two fine particulate matter standard reference materials (<4 μm and <10 μm) for the determination of organic and inorganic constituents. Anal. Bioanal. Chem..

[18] Valacchi G., Sticozzi C., Pecorelli A., Cervellati F., Cervellati C., Maioli E. (2012). Cutaneous responses to environmental stressors. Ann. N. Y. Acad. Sci..

[19] Magnani N.D., Muresan X.M., Belmonte G., Cervellati F., Sticozzi C., Pecorelli A., Miracco C., Marchini T., Evelson P., Valacchi G. (2016). Skin Damage Mechanisms Related to Airborne Particulate Matter Exposure. Toxicol. Sci..

[20] Bieber T. (2008). Atopic dermatitis. N. Engl. J. Med..

[21] Suárez-Fariñas M., Tintle S.J., Shemer A., Chiricozzi A., Nograles K., Cardinale I., Duan S., Bowcock A.M., Krueger J.G., Guttman-Yassky E. (2011). Nonlesional atopic dermatitis skin is characterized by broad terminal differentiation defects and variable immune abnormalities. J. Allergy Clin. Immunol..

[22] Gittler J.K., Shemer A., Suárez-Fariñas M., Fuentes-Duculan J., Gulewicz K.J., Wang C.Q., Mitsui H., Cardinale I., de Guzman Strong C., Krueger J.G. (2012). Progressive activation of T(H)2/T(H)22 cytokines and selective epidermal proteins characterizes acute and chronic atopic dermatitis. J. Allergy Clin. Immunol..

[23] Suárez-Fariñas M., Ungar B., Correa da Rosa J., Ewald D.A., Rozenblit M., Gonzalez J., Xu H., Zheng X., Peng X., Estrada Y.D. (2015). RNA sequencing atopic dermatitis transcriptome profiling provides insights into novel disease mechanisms with potential therapeutic implications. J. Allergy Clin. Immunol..

[24] Ewald D.A., Noda S., Oliva M., Litman T., Nakajima S., Li X., Xu H., Workman C.T., Scheipers P., Svitacheva N. (2017). Major differences between human atopic dermatitis and murine models, as determined by using global transcriptomic profiling. J. Allergy Clin. Immunol..

[25] Jin S.P., Li Z., Choi E.K., Lee S., Kim Y.K., Seo E.Y., Chung J.H., Cho S. (2018). Urban particulate matter in air pollution penetrates into the barrier-disrupted skin and produces ROS-dependent cutaneous inflammatory response in vivo. J. Dermatol. Sci..

[26] Li R., Ning Z., Majumdar R., Cui J., Takabe W., Jen N., Sioutas C., Hsiai T. (2010). Ultrafine particles from diesel vehicle emissions at different driving cycles induce differential vascular pro-inflammatory responses: Implication of chemical components and NF-kappaB signaling. Part. Fibre Toxicol..

[27] Totlandsdal A.I., Herseth J.I., Bølling A.K., Kubátová A., Braun A., Cochran R.E., Refsnes M., Ovrevik J., Låg M. (2012). Differential effects of the particle core and organic extract of diesel exhaust particles. Toxicol. Lett..

[28] Esser C., Rannug A. (2015). The aryl hydrocarbon receptor in barrier organ physiology, immunology, and toxicology. Pharmacol. Rev..

[29] Esser C., Bargen I., Weighardt H., Haarmann-Stemmann T., Krutmann J. (2013). Functions of the aryl hydrocarbon receptor in the skin. Semin. Immunopathol..

[30] Mitchell K.A., Elferink C.J. (2009). Timing is everything: Consequences of transient and sustained AhR activity. BioChem. Pharmacol..

[31] Furue M., Hashimoto-Hachiya A., Tsuji G. (2019). Aryl Hydrocarbon Receptor in Atopic Dermatitis and Psoriasis. Int. J. Mol. Sci..

[32] Köhle C., Bock K.W. (2007). Coordinate regulation of Phase I and II xenobiotic metabolisms by the Ah receptor and Nrf2. BioChem. Pharmacol..

[33] Nebert D.W., Roe A.L., Dieter M.Z., Solis W.A., Yang Y., Dalton T.P. (2000). Role of the aromatic hydrocarbon receptor and [Ah] gene battery in the oxidative stress response, cell cycle control, and apoptosis. BioChem. Pharmacol..

[34] Yu J., Luo Y., Zhu Z., Zhou Y., Sun L., Gao J., Sun J., Wang G., Yao X., Li W. (2019). A tryptophan metabolite of the skin microbiota attenuates inflammation in patients with atopic dermatitis through the aryl hydrocarbon receptor. J. Allergy Clin. Immunol..

[35] Tauchi M., Hida A., Negishi T., Katsuoka F., Noda S., Mimura J., Hosoya T., Yanaka A., Aburatani H., Fujii-Kuriyama Y. (2005). Constitutive expression of aryl hydrocarbon receptor in keratinocytes causes inflammatory skin lesions. Mol. Cell. Biol..

[36] Stockinger B., Di Meglio P., Gialitakis M., Duarte J.H. (2014). The aryl hydrocarbon receptor: Multitasking in the immune system. Annu. Rev. Immunol..

[37] Schiering C., Vonk A., Das S., Stockinger B., Wincent E. (2018). Cytochrome P4501-inhibiting chemicals amplify aryl hydrocarbon receptor activation and IL-22 production in T helper 17 cells. BioChem. Pharmacol..

[38] Anderson C., Hehr A., Robbins R., Hasan R., Athar M., Mukhtar H., Elmets C.A. (1995). Metabolic requirements for induction of contact hypersensitivity to immunotoxic polyaromatic hydrocarbons. J. Immunol..

[39] Davila D.R., Davis D.P., Campbell K., Cambier J.C., Zigmond L.A., Burchiel S.W. (1995). Role of alterations in Ca(2+)-associated signaling pathways in the immunotoxicity of polycyclic aromatic hydrocarbons. J. Toxicol. Environ. Health.

[40] Yamamoto O., Tokura Y. (2003). Photocontact dermatitis and chloracne: Two major occupational and environmental skin diseases induced by different actions of halogenated chemicals. J. Dermatol. Sci..

[41] Bonvallot V., Baeza-Squiban A., Baulig A., Brulant S., Boland S., Muzeau F., Barouki R., Marano F. (2001). Organic compounds from diesel exhaust particles elicit a proinflammatory response in human airway epithelial cells and induce cytochrome p450 1A1 expression. Am. J. Respir Cell Mol. Biol..

[42] Kepley C.L., Lauer F.T., Oliver J.M., Burchiel S.W. (2003). Environmental polycyclic aromatic hydrocarbons, benzo(a) pyrene (BaP) and BaP-quinones, enhance IgE-mediated histamine release and IL-4 production in human basophils. Clin. Immunol..

[43] Pei X.H., Nakanishi Y., Inoue H., Takayama K., Bai F., Hara N. (2002). Polycyclic aromatic hydrocarbons induce IL-8 expression through nuclear factor kappaB activation in A549 cell line. Cytokine.

[44] Matsuo K., Hatanaka S., Kimura Y., Hara Y., Nishiwaki K., Quan Y.S., Kamiyama F., Oiso N., Kawada A., Kabashima K. (2019). A CCR4 antagonist ameliorates atopic dermatitis-like skin lesions induced by dibutyl phthalate and a hydrogel patch containing ovalbumin. BioMed. Pharmacother..

[45] Kim S.R., Choi H.S., Seo H.S., Choi Y.K., Shin Y.C., Ko S.G. (2012). Topical application of herbal mixture extract inhibits ovalbumin- or 2,4-dinitrochlorobenzene-induced atopic dermatitis. Evid. Based Complement. Altern. Med..

[46] Kim D., Langmead B., Salzberg S.L. (2015). HISAT: A fast spliced aligner with low memory requirements. Nat. Methods.

[47] Pertea M., Pertea G.M., Antonescu C.M., Chang T.C., Mendell J.T., Salzberg S.L. (2015). StringTie enables improved reconstruction of a transcriptome from RNA-seq reads. Nat. Biotechnol..

[48] Pertea M., Kim D., Pertea G.M., Leek J.T., Salzberg S.L. (2016). Transcript-level expression analysis of RNA-seq experiments with HISAT, StringTie and Ballgown. Nat. Protoc..

