# Supplementary material for: Air Pollution and Atopic Dermatitis (AD): The Impact of Particulate Matter (PM10) on an AD Mouse-Model
Source: Int J Mol Sci. 2020 Aug 24;21(17):6079. doi: 10.3390/ijms21176079 (PMC7503766; doi:10.3390/ijms21176079)
Supplement: Supplementary file 1 [file ijms-21-06079-s001.pdf]

Air Pollution and Atopic Dermatitis (AD): The Impact of Particulate Matter (PM_{10}) on an AD Mouse-Model

Yu Ri Woo [1,†], Seo-Yeon Park [2,†], Keonwoo Choi [2], Eun Sun Hong [1], Sungjoo Kim [2] and Hei Sung Kim [1,2,*]

Supplementary Table S1. Top 50 significantly upregulated and downregulated genes (log_2 fold change ≥ 1.5 and adjusted *p*-value < 0.05) in each compared group

	PM vs. NC	Log_2 FC	AD vs. NC	Log_2 FC	AD + PM vs. NC	Log_2 FC	AD + PM vs. AD	Log_2 FC	AD + PM vs. PM	Log_2 FC	PM vs. AD	Log_2 FC
1	*Sprr2d*	4.537781	*Rnase2a*	15.353418	*S100a9*	175.102606	*S100a9*	21.032206	*S100a9*	43.232510	*Npy*	2.912855
2	*S100a9*	4.050253	*S100a9*	8.325451	*Sprr2d*	122.348283	*Sprr2b*	19.765663	*S100a8*	36.247161	*Fam3b*	2.237210
3	*Stfa3*	3.884888	*Sprr2d*	7.414729	*Sprr2b*	99.190350	*Saa3*	16.871093	*Saa3*	33.226551	*Guca2a*	2.082410
4	*Chil1*	3.852430	*Thrsp*	6.074408	*S100a8*	86.466724	*S100a8*	16.615182	*Sprr2b*	30.495545	*Wfdc3*	2.057602
5	*Dbp*	3.637145	*Sprr2a1*	5.429848	*Sprr2a3*	68.747026	*Sprr2d*	16.500708	*Sprr2d*	26.962138	*Il22ra2*	1.771707
6	*Il1b*	3.375845	*S100a8*	5.204079	*Serpinb3a*	65.385001	*Sprr2a3*	14.139278	*Sprr2a3*	21.554361	*Ugt1a1*	1.765702
7	*Sprr2a1*	3.293317	*Serpinb3a*	5.127475	*Stfa3*	51.600623	*Serpinb3a*	12.751891	*Serpinb3a*	21.542993	*Tesc*	1.728145
8	*Lce1h*	3.272727	*Sprr2b*	5.018316	*Sprr2a1*	50.181213	*Sprr2e*	12.506733	*Sprr2e*	16.809473	*Serpine2*	1.705879
9	*Sprr2b*	3.252618	*C1qtnf3*	4.536723	*Sprr2e*	43.315581	*Gm5416*	10.651026	*Gm5416*	16.545459	*Crabp1*	1.702428
10	*Lce1g*	3.202117	*Cxcl1*	3.865055	*BC100530*	29.494805	*Stfa3*	9.717934	*Sprr2a1*	15.237285	*Ptgs1*	1.689424
11	*Sprr2a3*	3.189472	*2610528A11Rik*	3.748969	*Saa3*	29.235396	*Sprr2a1*	9.241734	*Sprr2i*	13.596652	*Cbarp*	1.668524
12	*2610528A11Rik*	3.128210	*Angptl8*	3.702645	*BC117090*	27.688896	*Sprr2f*	8.996294	*Stfa3*	13.282395	*Adh6a*	1.641637
13	*Clca3a2*	3.088569	*Tnfaip6*	3.665933	*2610528A11Rik*	27.119970	*Sprr2i*	8.775266	*BC117090*	11.971038	*Gdpd2*	1.610049
14	*Lce1k*	3.049365	*Rgs1*	3.463642	*Sprr2i*	26.165916	*BC117090*	8.733096	*Sprr2f*	11.898848	*Rarres1*	1.602208
15	*Serpinb3a*	3.035094	*Sprr2e*	3.463381	*Gm5416*	25.737129	*Krt16*	8.550596	*Lcn2*	11.314357	*Ano7*	1.600783
16	*Thrsp*	3.008267	*Chil1*	3.413080	*Chil1*	23.799435	*Lcn2*	8.085627	*Krt6b*	10.906282	*Serpina3j*	1.593097
17	*Krtap21-1*	2.924621	*8430408G22Rik*	3.320192	*Sprr2f*	22.962500	*Krt6b*	7.671900	*BC100530*	10.128274	*Acoxl*	1.588232
18	*BC100530*	2.912126	*Ccl8*	3.262495	*Lcn2*	19.571697	*BC100530*	7.555283	*Lce3b*	9.559686	*Adh1*	1.587999
19	*Aox4*	2.876501	*Ccl7*	3.176686	*Stfa1*	17.513492	*Il1b*	7.414423	*Krt16*	9.550772	*Mt3*	1.565046
20	*Lce1f*	2.848601	*Cd163l1*	3.159971	*Rnase2a*	17.480094	*2610528A11Rik*	7.233981	*Ms4a6d*	9.338424	*Ndp*	1.560080
21	*Lce1i*	2.846397	*Ptx3*	3.071325	*Serpinb3b*	17.026990	*Chil1*	6.973008	*Stfa1*	8.717599	*Slc22a4*	1.531051
22	*Casp14*	2.835712	*Fcer1a*	3.066804	*Il1b*	15.898589	*Stfa1*	6.855981	*2610528A11Rik*	8.669484	*Tecr*	1.520385
23	*5830411N06Rik*	2.834779	*Tpsab1*	3.046646	*Cxcl1*	15.266021	*Lce3b*	6.312606	*Wfdc17*	8.488851	*Ano9*	1.514115
24	*Rgs1*	2.776930	*Sprr2i*	2.981781	*Krt16*	14.027059	*Serpinb3b*	6.150751	*Csf3r*	7.690534	*Hsd3b6*	1.501121
25	*Klk9*	2.747518	*Cxcr6*	2.929345	*Gsdmc*	13.943653	*Klk13*	6.027555	*Lce3a*	7.581050		
26	*Cd207*	2.600235	*Krtap21-1*	2.925668	*Retnlg*	13.784051	*Tmprss11g*	6.023283	*Ccl8*	7.572985		
27	*Sprr2e*	2.576855	*5830411N06Rik*	2.915997	*Klk13*	13.571149	*Lce3a*	5.917627	*Cxcl1*	7.504513		
28	*Chit1*	2.546383	*Igfbp2*	2.877333	*Lce3b*	12.008217	*Rptn*	5.506417	*Timp1*	7.101924		
29	*Aadacl3*	2.524152	*Tpsb2*	2.859246	*Lce3a*	11.751650	*Mmp3*	5.369114	*Retnlg*	6.997817		
30	*Lce1d*	2.513840	*Gsdmc*	2.839146	*Lce3f*	11.052984	*Lce3e*	5.314134	*Serpinb3b*	6.818736		
31	*Fam3b*	2.498548	*Mrap*	2.820249	*Mmp3*	10.906441	*Klk9*	5.298470	*Cd163*	6.801530		
32	*Serpinb3b*	2.497089	*Serpinb3b*	2.768278	*Ms4a6d*	10.838765	*Bcl2l15*	5.263128	*Slpi*	6.777415		
33	*8430408G22Rik*	2.491011	*Blk*	2.758426	*Rptn*	10.626554	*Slpi*	5.106645	*Mmp3*	6.745412		
34	*Calm4*	2.489697	*Il1f6*	2.677326	*Il1f6*	10.362620	*Gsdmc*	4.911213	*Ms4a4a*	6.573357		
35	*Cd3g*	2.488359	*Ccl12*	2.664043	*Ccl8*	9.964881	*Lce3f*	4.905179	*Mybpc2*	6.547380		
36	*Il1f6*	2.459084	*Retnlg*	2.652207	*Klk9*	9.784243	*Teddm3*	4.901073	*Sell*	6.337477		
37	*Klk8*	2.456366	*Lce1h*	2.649690	*Krt6b*	9.746359	*Timp1*	4.841521	*Chil1*	6.177772		

38	*Apoc4*	2.416382	*Ces1f*	2.632184	*Slpi*	9.610843	*Ms4a6d*	4.839326	*Klk13*	6.164222
39	*Gsdmc*	2.414530	*Cd3g*	2.632181	*Csf3r*	9.543424	*Csf3r*	4.739358	*Cd300ld3*	6.054850
40	*Tmprss4*	2.403543	*Lce1g*	2.625508	*Lce3e*	9.465151	*Ms4a4a*	4.661390	*Rptn*	6.011338
41	*Lce1c*	2.402870	*Eln*	2.622964	*Timp1*	9.302482	*Clec4d*	4.294255	*Ccl6*	5.995977
42	*Nppb*	2.390614	*Cpa3*	2.616742	*Wfdc17*	8.724972	*Cd300ld3*	4.231297	*Mrc1*	5.794368
43	*S100a8*	2.385476	*Ccl11*	2.578108	*Clec4d*	8.340879	*Sell*	4.101222	*Gsdmc*	5.774894
44	*Lce1a2*	2.369149	*Hsd11b1*	2.560542	*Teddm3*	8.261515	*Il1f9*	4.090919	*Fcgr2b*	5.765075
45	*Dusp1*	2.366247	*Bcl2a1b*	2.559817	*Sell*	8.249449	*Gsdmc2*	4.004163	*Ccr1*	5.588996
46	*Cyp1a1*	2.364800	*Fasn*	2.553306	*Gsdmc2*	8.046147	*Rnase2b*	3.941229	*Lce3f*	5.526953
47	*Lce1e*	2.351248	*Mcpt4*	2.552227	*Bcl2l15*	8.039882	*Cxcr2*	3.934086	*Rnase2b*	5.485106
48	*Lce1b*	2.344811	*Cd209e*	2.480916	*Ms4a4a*	8.018789	*Il1f6*	3.870511	*Ms4a6c*	5.451113
49	*Cxcr6*	2.343134	*Cma1*	2.449102	*Tmprss11g*	7.967437	*Ifitm1*	3.852028	*Rnase2a*	5.250776
50	*Oas1f*	2.326978	*Ptges*	2.418173	*Cd163*	7.650302	*Fetub*	3.822046	*Tmprss11g*	5.243561
Other	*Klk13*	2.2016	*Ccl2*	2.414083	*Fasn*	2.203831				
	Cxcl1	2.034245	*Gm436*	2.385544	*Nfkbid*	1.785860				
	Krt1	2.012479	*Aox4*	2.30949	*Nfkb1*	1.724704				
	Il-22	1.980533	*Klk13*	2.251518	*Scd1*	1.718452				
	Nfkbia	1.85684	*Ms4a6d*	2.239726						
	Krt10	1.563309	*Ctsk*	2.227424						
	Scd1	1.556242	*Lilr4b*	2.2165						
	Msmo1	1.555874	*Lilr4b*	2.2165						
	Il-18	1.545413	*Mmp3*	2.03133						
			Gsdmc2	2.009446						
			Pdss1	2.009415						
			Aadacl3	2.006186						
			Col6a2	1.975488						
			Lox	1.970348						
			Il1f8	1.930127						
			Rptn	1.929849						
			Col6a1	1.91373						
			Mrgprb1	1.911956						
			Mmp19	1.909659						
			Sgpp2	1.874239						
			Adam12	1.872757						
			Lce1f	1.856882						
			Degs2	1.851576						
			Mrc1	1.847883						
			Gpr65	1.733434						
			Lrcc17	1.725056						
			Ms4a4a	1.720257						
			Acot5	1.71783						

Col6a3	1.70334
Prss27	1.680144
Aadac	1.671635
Ms4a2	1.644655
Slc5a1	1.619429
Elovl3	1.605484
Cyp7b1	1.576083
Cdh11	1.557458
